# ST-Segment Elevation in the Setting of Diabetic Ketoacidosis: Is It Acute Coronary Syndrome?

**DOI:** 10.7759/cureus.7409

**Published:** 2020-03-25

**Authors:** Jesse Wray, Michael J Yoo, Rachel E Bridwell, Lloyd Tannenbaum, Jonathan Henderson

**Affiliations:** 1 Emergency Medicine, Brooke Army Medical Center, Fort Sam Houston, USA; 2 Emergency Medicine, San Antonio Uniformed Services Health Education Consortium, San Antonio, USA

**Keywords:** diabetic ketoacidosis, st-segment elevation, hyperkalemia, pseudo-infarct pattern

## Abstract

Diabetic ketoacidosis (DKA) with resulting hyperkalemia can lead to ST-segment elevations on electrocardiogram (ECG). Previous publications theorize that significant improvements in patient potassium levels lead to the resolution of this rare phenomenon, also known as "pseudo-infarct" pattern. The authors provide a unique case along with a literature review of DKA-associated ST-segment elevations. This specific case distinctively demonstrates the resolution of the pseudo-infarct pattern in the setting of minor improvements in serum potassium and continued acidosis.

## Introduction

Diabetic ketoacidosis (DKA) is one of the most serious complications of diabetes mellitus, characterized by hyperglycemia, metabolic acidosis, ketonemia, and electrolyte abnormalities such as hyperkalemia [1]. Classically, hyperkalemia-related electrocardiogram (ECG) changes include PR interval prolongation, peaked T-waves, widening of the QRS complex, and a sine-wave pattern at severely elevated levels [2]. Less commonly, hyperkalemia can lead to ST-segment elevation, especially in the setting of DKA, though the exact etiology of this injury pattern is likely multifactorial [3-4]. Previous case reports document resolution of this injury pattern with the correction of hyperkalemia. The following case demonstrates evolving ST-segment elevations in a patient with DKA that resolved with only minor improvements in his serum potassium. In contrast, previous publications suggest more significant corrections are necessary for ST-segment elevation resolution.

## Case presentation

A 19-year-old male with a past medical history of type 1 diabetes mellitus presented to the emergency department (ED) with one day of nausea, vomiting, and abdominal pain. On arrival to the ED, the patient’s vital signs were: temperature of 37 °C, heart rate of 126 beats per minute, blood pressure of 122/55 mmHg, respiratory rate of 40 breaths per minute, and pulse oximetry of 100% on room air. On review of systems, he described nausea, diffuse back pain, and generalized abdominal pain but denied polyuria, polydipsia, or recent weight loss. Physical exam revealed a diffusely tender abdomen without rebound or guarding and diffuse back tenderness without gross deformities or evidence of trauma.

**Figure 1:**
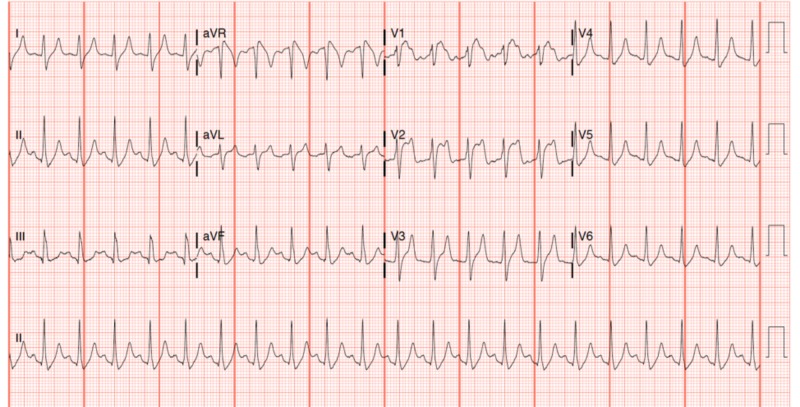
Initial electrocardiogram obtained upon arrival to the emergency department demonstrating ST-segment elevation in leads V1 and V2

Serum studies demonstrated a pH of 7.09, bicarbonate of 9.7 mmol/L, anion gap of 27, potassium of 5.7 mEq/L, glucose that exceeded the laboratory’s maximum measurable value of 700, and an undetectable troponin. An ECG obtained on arrival demonstrated 3 mm of ST-segment elevation in V1, 5 mm of ST-segment elevation in V2, and sinus tachycardia to 127 (Figure 1). Intravenous access (IV) was established, and the patient received a 1 L bolus of lactated ringer’s solution and was started on a regular insulin drip at 0.1 U/kg/h with no initial bolus.

**Figure 2:**
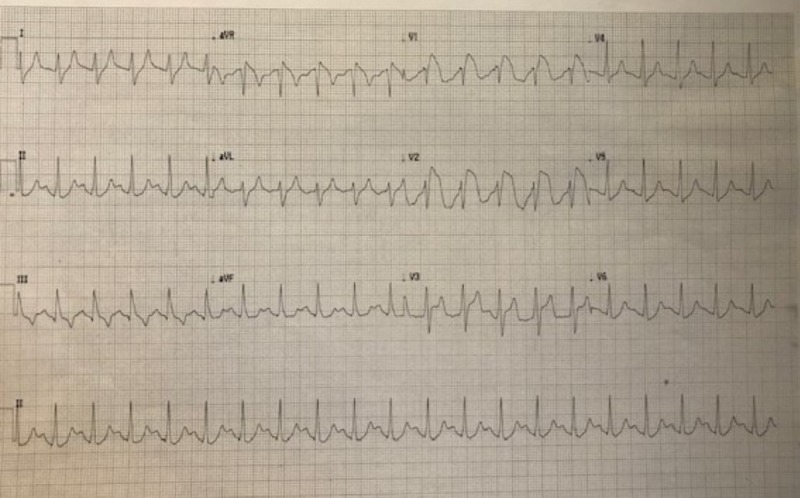
Increasing ST-segment elevations in leads V1 and V2; new ST-segment elevations in lead V3

**Figure 3:**
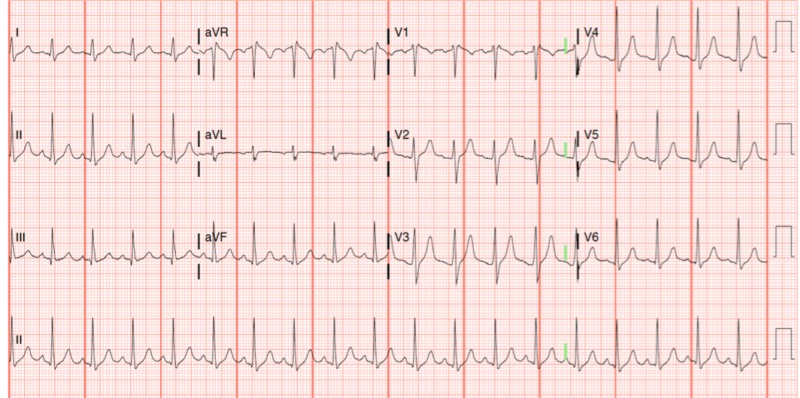
Post-catheterization electrocardiogram with resolution of the ST-segment elevations

A repeat ECG obtained 30 minutes later demonstrated interval progression of the ST-segment elevations in V1, V2, and V3 (Figure 2). Based on these dynamic changes, cardiology recommended cardiac catheterization which revealed normal coronary arteries with TIMI (Thrombolysis in myocardial infarction) III flow as well as no evidence of coronary artery dissection or vasospasm. A post-catheterization ECG demonstrated a complete normalization of the ST-segments (Figure 3). Repeat laboratory testing upon completion of his catheterization resulted in a pH of 7.09, glucose of 619 mg/dL, and potassium of 5.34 mEq/L. The troponin level following cardiac catheterization became detectable with a peak level of 0.098, consistent with a type IV myocardial infarction [5]. The patient was admitted to the medical intensive care unit (MICU) and discharged 3 days later with an uncomplicated hospital course.

## Discussion

Hyperkalemia-associated ST-segment elevations in the setting of DKA is a known but rare phenomenon. A literature review identified 19 documented cases of these events (Table 1) [1-4,6-20]. In all cases with documented delta potassium, ST-segment elevations resolved with potassium correction and continued treatment of DKA. During the initial ST-segment elevations, the mean potassium was 7.47 mEq/L (standard deviation [SD] = 1.2), and the mean pH was 7.05 (SD = 0.13). At the time of ST-segment normalization, the mean serum potassium was 4.88 mEq/L (SD = 0.89), and the mean pH was 7.25 (SD = 0.23). However, the true value in interpreting this data as a whole is limited given incomplete reporting of data in prior cases. Many of these reports theorize that the resolution of these ECG findings correlated with significant corrections in patient potassium levels [3,6-8,10-11]. This specific case demonstrated normalization of the patient’s ECG with clinically insignificant improvement in serum potassium levels. Specifically, this patient’s potassium decreased by 6.3% (5.7 mEq/L to 5.34 mEq/L) compared to a mean decrease of 34.7% in prior cases. The laboratory-specific margin of error for serum potassium was reported to be 0.1 mEq/L which shows that this change in potassium was essentially negligible. Additionally, the rapid, dynamic changes in this patient’s ECG have not been previously described. Prior case reports discuss DKA-associated metabolic acidosis may also contribute to ST-segment elevation [2-4]. However, our case did not have significant changes in pH, serum bicarbonate, or other serum electrolytes with ECG normalization. The exact etiology of the ST-segment elevations, in this case, remains likely multifactorial, and further research is needed to better clarify this phenomenon. Given the lack of coronary artery disease noted on angiography, the coved ST-segment elevations were likely secondary to the known metabolic derangements associated with DKA. However, the authors theorize that even minor improvements in patient potassium levels may lead to normalization of ST-segment changes on ECG.

**Table 1:** Summary of previously published pseudo-infarct pattern electrocardiograms in the setting of diabetic ketoacidosis associated hyperkalemia. Initial and subsequent potassium, pH, and glucose levels are given. Abbreviations: M = Male, F = Female, K+ = Potassium, NR = Not Reported, NA = Not Available.

Case	Age	Sex	Initial K+	Initial pH	Initial glucose	End K+	End pH	End glucose	Delta K+	Delta pH
Sharma [6]	43	M	8.1	7.23	786	NR	NR	NR	NA	NA
Bellazzini [7]	40	M	8.3	7.01	1818	7.3	NR	NR	1	NA
Sweterlitsch [3]	46	M	7.9	6.97	1586	3.8	7.12	858	4.1	0.15
Ziakas [8]	33	F	7.2	7.16	High	4.9	NR	255	2.3	NA
Wang [9]	38	M	7.9	7.21	839	5.1	NR	NR	2.8	NA
Aksakal [4]	58	M	4.4	7.15	712	NR	7.34	263	NA	0.19
Moulik [10]	42	M	8.9	7.06	High	4.7	NR	NR	4.2	NA
Cohen [2]	38	M	7.5	6.94	1206	3.8	7.43	350	3.7	0.49
Simon [11]	59	M	8.1	7.06	1644	5.4	NR	NR	2.7	NA
Carrizales-Sepúlveda [1]	48	M	5.7	6.94	620	NR	NR	NR	NA	NA
Egred [12]	30	M	6.9	NR	NR	NR	NR	NR	NA	NA
Ruiz-Morales [13]	47	F	6.7	NR	985	4.6	NR	613	2.1	NA
Sims [14]	20	M	9.4	6.92	1240	5.7	NR	NR	3.7	NA
Chawla [15]	48	M	8.3	7.09	840	4	7.44	452	4.3	0.35
Lim [16]	59	F	7.2	6.74	1020	4.8	6.9	792	2.4	0.16
Kamimura [17]	45	M	7.3	7.01	1827	5.1	NR	NR	2.2	NA
Johnson [18]	48	M	5.8	7.17	750	NR	NR	NR	NA	NA
Gelzayd [19]	42	M	8.7	NR	1240	4.4	NR	NR	4.3	NA
Tatli [20]	20	M	7.7	7.1	740	4.7	NR	NR	3	NA

## Conclusions

Emergency physicians are faced with diagnostic dilemmas on a frequent basis. One such conundrum is ruling out myocardial infarction in the setting of DKA. The above case and associated literature review highlight the diagnostic obfuscation from metabolic derangements associated with DKA mimicking injury pattern on ECG. Clinicians must continue to recognize that myocardial infarction is a known precipitant of DKA, and these two pathologies can coexist. However, this case report and literature review reinforce that DKA can be associated with ECG manifestations mimicking acute myocardial ischemia. Emergency medicine providers should be aware of this phenomenon in order to attempt to differentiate it from an ST-segment elevation myocardial infarction. 
